# Synergistic and antagonistic interactions between antibiotics and synbiotics in modifying the murine fecal microbiome

**DOI:** 10.1007/s00394-019-02035-z

**Published:** 2019-07-01

**Authors:** Angela Jačan, Karl Kashofer, Geraldine Zenz, Esther E. Fröhlich, Florian Reichmann, Ahmed M. Hassan, Peter Holzer

**Affiliations:** 1 CBmed GmbH-Center for Biomarker Research in Medicine, Stiftingtalstrasse 5, 8010 Graz, Austria; 2 Diagnostic and Research Institute of Pathology, Diagnostic and Research Center of Molecular Biomedicine, Medical University of Graz, Neue Stiftingtalstrasse 6, 8010 Graz, Austria; 3 Research Unit of Translational Neurogastroenterology, Division of Pharmacology, Otto Loewi Research Center, Medical University of Graz, Universitätsplatz 4, 8010 Graz, Austria

**Keywords:** Antibiotics, Bacterial richness, Bacterial diversity, Bacterial orders, Gut microbiota, Recovery from antibiotic treatment, Synbiotics

## Abstract

**Purpose:**

Pro- and synbiotics have been reported to ameliorate the adverse (dysbiotic) effects of antibiotics on the gut microbial architecture, but little is known how synbiotics and antibiotics interact with each other in shaping the gut microbiota. To explore this mutual interaction we examined, first, the effect of a multi-strain synbiotic on antibiotic-induced dysbiosis and, second, the dysbiotic effect of antibiotics followed by prolonged synbiotic exposure.

**Methods:**

The synbiotic containing nine bacterial strains was administered to male mice via the drinking water, while the antibiotic mix containing bacitracin, meropenem, neomycin, and vancomycin was administered via oral gavage. Two experimental protocols were used. In protocol 1, mice were administered placebo or synbiotic for 3 weeks prior to and during an 11-day vehicle or antibiotic treatment. In protocol 2 the synbiotic was administered for a prolonged period of time, starting 3 weeks prior and continuing for 12 weeks after an 11-day vehicle or antibiotic treatment. Subsequently, the fecal microbiome was analyzed by 16S rRNA sequencing using oligonucleotide primers 16s_515_S3_fwd: GATTGCCAGCAGCCGCGGTAA and 16s_806_S2_rev: GGACTACCAGGGTATCTAAT followed by sequencing using the Ion Torrent One. The final sequence files were analyzed by QIIME 1.8 workflow scripts.

**Results:**

Antibiotic treatment markedly decreased the bacterial richness and diversity of the fecal microbiota. Synbiotic administration for 3 weeks prior to and during an 11-day antibiotic treatment preserved the Lactobacillales and expanded the Verrucomicrobiales and Bifidobacteriales order, but did not prevent the depletion of Bacteroidales and the short-term proliferation of Enterobacteriales. When the synbiotic administration was continued for 12 weeks after the end of antibiotic treatment, the rise of Verrucomicrobiales was maintained, whereas the preservation of Lactobacillales and boost of Bifidobacteriales was lost. The abundance of Clostridiales was enhanced by long-term synbiotic treatment after short-term exposure to antibiotics, while the antibiotic-depleted Bacteroidales underwent a delayed recovery.

**Conclusions:**

There are complex synergistic and antagonistic interactions of synbiotics and antibiotics in influencing distinct bacterial orders of the fecal microbiota. The impact of a short-term antibiotic exposure is profoundly different when analyzed after synbiotic pretreatment or following prolonged synbiotic administration in the post-antibiotic period.

## Introduction

Bacteria represent a major constituent of the gut microbiota, the major phyla in the mammalian intestine being Bacteroidetes, Firmicutes, Proteobacteria, Actinobacteria and Fusobacteria [1, 2]. The richness and diversity of the human gut microbiota is influenced by many factors including age, diet, medications (especially antibiotics), environmental factors and host genetics [2–8]. As long as the diversity of species is high, the intestinal microbiota is thought to have a beneficial impact on the host, ranging from the production of vitamins of the B and K groups to metabolism of many dietary substrates including polyphenols and otherwise indigestible plant-derived fibers, the latter being converted to short-chain fatty acids [2, 4, 9, 10]. Microbial metabolites derived from dietary substrates exert a variety of effects on the host by modulating the function of the gastrointestinal, immune, metabolic, cardiovascular and nervous system [3, 4, 9–14]. Gut dysbiosis becomes manifest when the species richness and diversity of the intestinal microbiota are disrupted. This condition is thought to have an impact on many diseases as diverse as inflammatory bowel disease [15], obesity and metabolic syndrome [3, 16, 17], non-alcoholic fatty liver disease [18] as well as neurologic and psychiatric disorders [11–13].

Against this background, pre-, pro- and synbiotics have gained increasing attention for their potential in the management of microbiome-related diseases. Probiotics are defined as “live microorganisms which when administered in adequate amounts confer a health benefit on the host” [19, 20]. Presently, they comprise mostly lactic acid-producing bacteria that belong to the genera *Lactobacillus* and *Bifidobacterium*. Prebiotics are dietary components that aid the growth of beneficial microorganisms [4, 21] while synbiotics represent combinations of pre- and probiotics. A vast number of studies suggest that probiotics have a beneficial influence on various pathological conditions ranging from gastroenterological, immunological and metabolic to neuropsychiatric diseases, however with inconsistent outcomes [20]. The health-promoting effects of probiotics are thought to arise from several mechanisms: colonization of the gut, improvement of the microbiota profile if dysbiosis is present, enforcement of the intestinal mucosal barrier and promotion of immune homeostasis [7, 19, 22–24]. For instance, various species of the genus *Lactobacillus* have a beneficial effect in experimental models of colitis [25] as well as in necrotizing enterocolitis and acute pediatric gastroenteritis [26, 27].

Probiotics have long been considered to represent an adjunct therapy to ameliorate the adverse effects of antibiotic treatment on the gut microbiota, which may result in dysbiosis and antibiotic-associated diarrhea. Although this promise has not been fulfilled in some human studies [28], an overview of the available evidence from human studies indicates that particular probiotics can prevent or reduce antibiotic-associated diarrhea [29–32]. There are also some human trials to suggest that multi-strain probiotics may be of superior efficacy over mono-strain probiotics [26, 31]. On the other hand, there is evidence that probiotics colonizing the gut may rather impair than aid the reconstitution of a normal gut microbiota after antibiotic treatment of humans [7].

Collectively, these findings highlight a lack of information about the mutual interaction between antibiotics and probiotics in shaping the architecture of the gut microbiota. It is therefore difficult to make full use of the therapeutic potential of probiotics/synbiotics in the prevention or treatment of antibiotic-induced adverse reactions such as antibiotic-associated diarrhea. Specifically, little is known as to how, on the one hand, synbiotics modify the ability of antibiotics to cause dysbiosis and whether, on the other hand, any short- or long-term effect of antibiotics is influenced by the presence of probiotics or synbiotics. The aim of our study was to address these questions in mice, using a combination of four non-absorbable antibiotics (bacitracin, meropenem, neomycin and vancomycin) administered via gastric gavage and a synbiotic containing nine strains of probiotic bacteria administered via the drinking water. In protocol 1, the effect of this multi-strain synbiotic to alleviate antibiotic-induced dysbiosis was examined, whereas in protocol 2 the effect of the antibiotics in the presence of the synbiotic was tested. Using 16S rDNA sequencing, we obtained an unbiased view of the fecal microbiome profile, its perturbation by gut-directed antibiotic treatment and its modification by the synbiotic.

The synbiotic chosen for this study (marketed as OMNi BiOTiC^®^ STRESS Repair and Ecologic^®^825) has thus far been studied in humans only. Oral intake of the synbiotic by healthy volunteers for 4 weeks failed to change microbial diversity in stool samples, but enlarged the abundance of *Bacteroides* sp. and *Alistipes* sp. [33]. A similar finding was obtained after 4 weeks of synbiotic administration to patients with diarrhea-predominant irritable bowel syndrome (IBS-D) while the abundance of unclassified *Lactobacillaceae* was increased [34]. In contrast, the microbial diversity in gastric and duodenal biopsies of IBS-D patients was significantly enhanced by the synbiotic [34]. Despite the small effect on the microbial profile in feces, prolonged synbiotic intake (4 weeks or more) improved gut barrier function in IBS-D patients [34], patients with severe pouchitis associated with ulcerative colitis [35] as well as patients with Alzheimer’s disease [36] and reduced symptom severity in IBS-D patients [34]. Furthermore, the synbiotic altered resting-state functional connectivity in the brain of healthy volunteers [37], which was associated with distinct alterations in brain activation patterns in response to emotional memory and emotional decision-making tasks [33]. In addition, the synbiotic reduced cognitive reactivity to sad mood in healthy volunteers [33] and patients with euthymic bipolar disorder [38].

## Methods

### Ethics statement

The experimental procedures and the number of animals used were approved by the ethical committee at the Federal Ministry of Science, Research and Economy of the Republic of Austria (BMWFW-66.010/0050-WF/V/3b/2017), and conducted according to the Directive of the European Parliament and of the Council of September 22, 2010 (2010/63/EU). The experiments were designed in such a way that both the number of animals used and their suffering was minimized.

### Experimental animals

The experiments were carried out with adult, 8 weeks old, male C57BL/6N mice obtained from Charles River Laboratories (Sulzfeld, Germany). Throughout the experiments the mice were kept in groups of two. The housing conditions including lighting (12-h light/dark cycle, maximal light intensity 100 lx), temperature (set point 22 °C), and relative air humidity (set point 50%) were tightly controlled. Tap water and standard laboratory chow were provided ad libitum.

### Synbiotic source, dosage and treatment

The synbiotic (marketed as OMNi BiOTiC^®^ STRESS Repair by Institut Allergosan, Graz, Austria, and as Ecologic^®^825 by Winclove, Amsterdam, The Netherlands) and placebo were kindly provided as water-soluble preparations by Winclove. The synbiotic consisted of 9 bacterial strains (*Lactobacillus casei W56*, *Lactobacillus acidophilus W22*, *Lactobacillus paracasei W20*, *Bifidobacterium lactis W51*, *Lactobacillus salivarius W24*, *Lactococcus lactis W19*, *Bifidobacterium lactis W52*, *Lactobacillus plantarum W62* and *Bifidobacterium bifidum W23*). Besides the bacterial strains (1.9%), the synbiotic contained maize starch (62.0%), maltodextrin (15.5%), inulin P7 (13.8%), potassium chloride (3.4%), magnesium sulfate (1.6%), fructooligosaccharides P7 (1.2%), enzymes (0.5%) and manganese sulfate (0.0035%). The placebo added to the drinking water included maize starch (76%), maltodextrin (19%) as the main carriers [33, 39] as well as potassium chloride (3.4%), magnesium sulfate (1.6%) and manganese sulfate (0.0035%). The synbiotic was dissolved in fresh tap water to contain the bacterial strains at a concentration of 2.5 × 10^9^ CFU/l.

Beginning at the age of 8 weeks, mice received the synbiotic or placebo via the drinking water, and during the whole experiment the synbiotic or placebo solution was the only source of water. The experiments of protocol 1 (see below), in which the intake of the synbiotic and placebo solution per cage was monitored for 3 weeks, showed that the intake of the synbiotic solution was nominally, but not significantly, higher than the intake of the placebo solution. The synbiotic and placebo solutions were prepared fresh on Mondays, Wednesdays and Fridays. The survival of the probiotic strains after 48 h in tap water was confirmed in two cell count/growth tests by plating the synbiotic at 1:10 000 dilution on De Man, Rogosa and Sharpe agar. In the first test the CFU/plate count increased from 128 to 176 and in the second test the CFU/plate count increased from 160 to 240 during the 48-h test period.

### Antibiotic sources, dosages and treatment

A combination of four non-absorbable antibiotics with a spectrum of action that affects a broad range of intestinal bacteria was used to deplete the gut microbiota. The antibiotics used included bacitracin (bacitracin from *Bacillus licheniformis,* catalogue number 11702, Sigma-Aldrich, Vienna, Austria), meropenem (Optinem^®^, Astra Zeneca Österreich GmbH, Vienna, Austria), neomycin (neomycin trisulfate salt hydrate, catalogue number N5285, Sigma-Aldrich) and vancomycin (vancomycin hydrochloride from *Streptomyces orientalis,* catalogue number 4747, Sigma-Aldrich). The ability of this antibiotic combination plus ampicillin to deplete the gut microbiota has previously been demonstrated [40]. Ampicillin was excluded from the combination used here because it is absorbed from the gut [40] and could have confounding systemic effects. The antibiotics (pH 6.98–7.14) or vehicle (distilled water) were administered by oral gavage at a volume of 10 ml/kg. For each gavage session, the antibiotics (109.0 mg bacitracin, 108.0 mg neomycin, 21.6 mg meropenem, and 6.48 mg vancomycin) were dissolved in 4.5 ml of distilled water. The dosing of antibiotics was based on previous studies [40]. The administration of the antibiotics by gavage and of the synbiotic and placebo via the drinking water was chosen to avoid any chemical interaction between the compounds that may occur if they were administered in a common solution.

At the age of 11 weeks, mice were treated with the combination of the four antibiotics or vehicle by oral gavage once daily for 11 days to ensure a steady dosing of the animals with the antibiotics. The antibiotic mixture was administered approximately 1 h before the onset of the dark phase. Each day the mice were weighed before gavage, and the gavage volume was adjusted accordingly. Because of their coprophagic behavior, all cage mates received the same intervention (antibiotic combination or vehicle).

### Experimental protocols

**Fig. 1:**
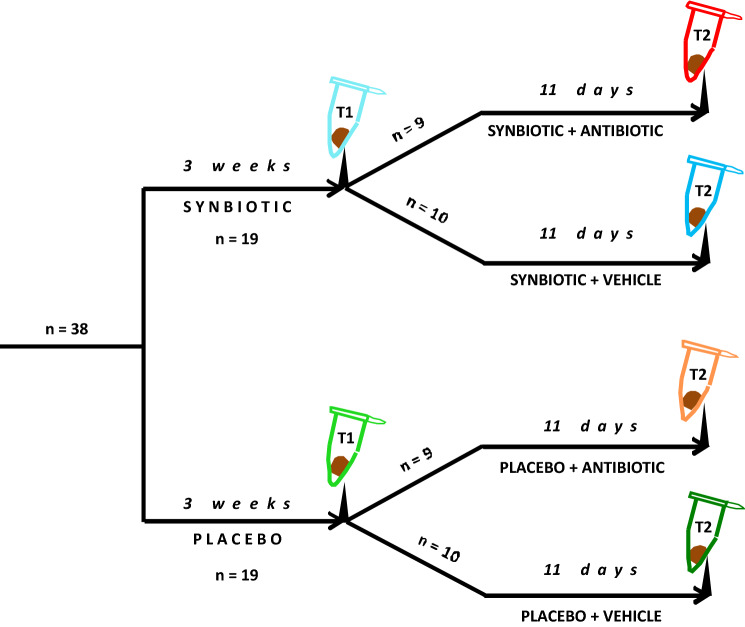
Protocol 1. Mice were treated with placebo or the synbiotic, followed by treatment with vehicle or the antibiotic combination, for the periods indicated. T1 and T2 mark the time points when fecal pellets were harvested for microbiome analysis. The number of animals used in each experimental group is also indicated

**Fig. 2:**
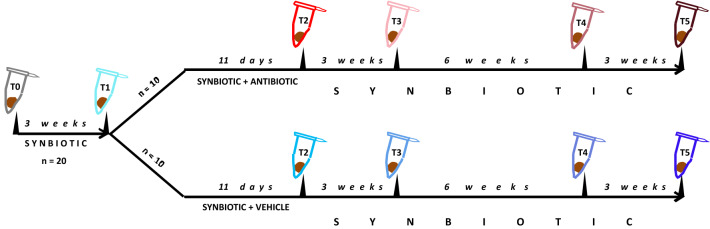
Protocol 2. Mice were treated with the synbiotic, followed by treatment with vehicle or the antibiotic combination, for the periods indicated. T0–T5 mark the time points when fecal pellets were harvested for microbiome analysis. The number of animals used in each experimental group is also indicated

The interaction between synbiotic and antibiotic treatment in modifying the gut microbiota was investigated with two experimental protocols (Figs. 1, 2). In both protocols the mice were randomly allocated to the different intervention groups.

In protocol 1, mice were administered placebo or the synbiotic for 3 weeks prior to the administration of vehicle or the antibiotic combination for 11 days (Fig. 1). The experiment ended on day 11 of the antibiotic treatment. Fecal pellets for microbiome analysis were harvested immediately before and at the end of antibiotic treatment period in a total of 4 experimental groups: placebo + vehicle, placebo + antibiotics, synbiotic + vehicle and synbiotic + antibiotics (Fig. 1).

In protocol 2, mice were administered the synbiotic for 3 weeks prior to the administration of vehicle or the antibiotic combination for 11 days similarly to one arm of protocol 1 (Fig. 2). However, in protocol 2, the administration of the synbiotic was continued during treatment with vehicle or the antibiotic combination for a period of 12 weeks after the treatment with vehicle or the antibiotic combination had been completed. Fecal pellets for microbiome analysis were harvested at six time points throughout the experiment in a total of two experimental groups: synbiotic + vehicle, synbiotic + antibiotics (Fig. 2).

### Harvesting of feces

At different time points of the experiment (Figs. 1, 2), fecal pellets were harvested by placing the mice in separate cages for up to 5 min and collecting the stool pellets produced. The freshly collected pellets were placed in Eppendorf tubes and frozen within minutes. The fecal samples were stored at − 70 °C until analysis.

### Microbiome analysis

Bacterial DNA was extracted from single fecal pellets with the Maxwell RSC Blood DNA Kit (Promega, Mannheim, Germany) according to the manufacturer’s instructions with slight modifications. Using the lysis buffer, stool samples were homogenized on a MagNA Lyser Instrument using MagNA Lyser Green Beads (Roche Diagnostics GmbH, Mannheim, Germany). After homogenization, the samples were treated with 2.5 mg/ml lysozyme (Roth GmbH, Karlsruhe, Germany) for 30 min at 37 °C, followed by digestion with 1 mg/ml proteinase K for 60 min at 56 °C. The enzyme was inactivated by exposure to 95 °C for 10 min, after which 600 µl of lysate was used for DNA isolation in the Maxwell RSC. The DNA concentration was measured by Picogreen fluorescence.

Bacterial 16S rRNA was amplified with the Mastermix 16S Complete PCR kit (Molzym, Bremen, Germany) according to the manufacturer’s instructions using a 0.4 µM final concentration of primers and 57 °C as annealing temperature for 25 cycles. The first PCR reaction product was subjected to a second round of PCR with primers fusing the 16S primer sequence to the A and P adapters necessary for Ion Torrent sequencing, while additionally a molecular barcode sequence was included to allow multiplexing of up to 96 samples simultaneously. PCR products were subjected to agarose gel electrophoresis, and the band of the expected length (350 nt) was excised from the gel and purified using the QiaQuick (Qiagen, Hilden, Germany) gel extraction system. The DNA concentration of the final PCR product was measured by Picogreen fluorescence.

### Sequencing

Amplicons from up to 60 samples were pooled at equimolar amounts and subjected to emulsion PCR using the Ion Torrent One Touch 2.0 Kit according to the manufacturer’s protocols. After emulsion PCR the beads were purified on the Ion ES station and loaded onto Ion Torrent 318 chips for sequencing. Sequencing reactions were performed on Ion Torrent PGM using the Ion 400BP Sequencing Kit running for 1000 flows (all reagents from Thermo Fisher Scientific, Waltham, MA, USA). Sequences were split by barcode and transferred to the Torrent Suite server. Unmapped bam files were used as input for bioinformatics.

### Bioinformatics and phylogenetic analysis

All sequences were initially trimmed by a sliding window quality filter with a width of 20 nt and a cutoff of Q20. Reads shorter than 100 nucleotides and reads mapping to the human genome were removed using DeconSeq [41]. The resulting reads were subjected to error correction using the Acacia tool [42] leading to error correction of 10–20% of the reads. Subsequently, PCR chimeras were removed by the USEARCH algorithm in de novo and reference-based settings [43]. The final sequence files were analyzed by QIIME 1.8 workflow scripts [44]. OTU search was performed using the parallel_pick_open_reference_otus workflow script and the greengenes 13_8 reference database.

### Statistical analysis and visualization

Results were statistically evaluated either with SPSS 22 (SPSS Inc., Chicago, IL, USA), R (R Development Core Team 2011) (v 3.2.1, packages stats, missMDA, nlme) using Tibco^®^ Spotfire^®^ (v 7.0.0) or Prism 8 GraphPad. For microbiome analysis OTUs were visualized as OTU tables, bar charts and PCoA plots using the QIIME core microbiome script, and subsequently color-coded and formatted in Inkscape. Additionally, groupings supplied in the mapping file were tested for statistical significance using the QIIME implementation of the Adonis test. The significance of individual bacterial strains was determined by Kruskal–Wallis test. Linear discriminant analysis (LDA) effect size (LEfSe) analysis [45] was performed to detect statistically relevant strains in the study groupings. Time- and treatment-related shifts in bacterial taxa were evaluated if the relative abundance of a bacterial order reached at least 2% in any of the samples. Differences in the relative abundances of bacterial orders in the selected samples were analyzed with the Prism 8 GraphPad software. In protocol 1, two-way ANOVA followed by Tukey’s HSD post hoc test was used, while in protocol 2 two-way repeated measures ANOVA followed by Sidak’s multiple comparison test was employed. *p* values < 0.05 were regarded as significant.

## Results

### Effects of synbiotic and antibiotic treatment on fecal microbiome in protocol 1

**Fig. 3:**
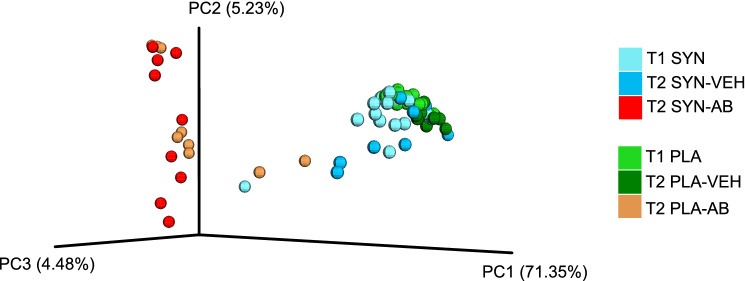
Principal coordinate analysis (PCoA) plot based on weighted UniFrac distance between samples obtained in protocol 1. T1 and T2 mark the time points when fecal pellets were harvested for microbiome analysis (see Fig. 1). Treatments: PLA, placebo (for synbiotic); SYN, synbiotic; VEH, vehicle (for antibiotic treatment); AB, antibiotic combination

**Fig. 4:**
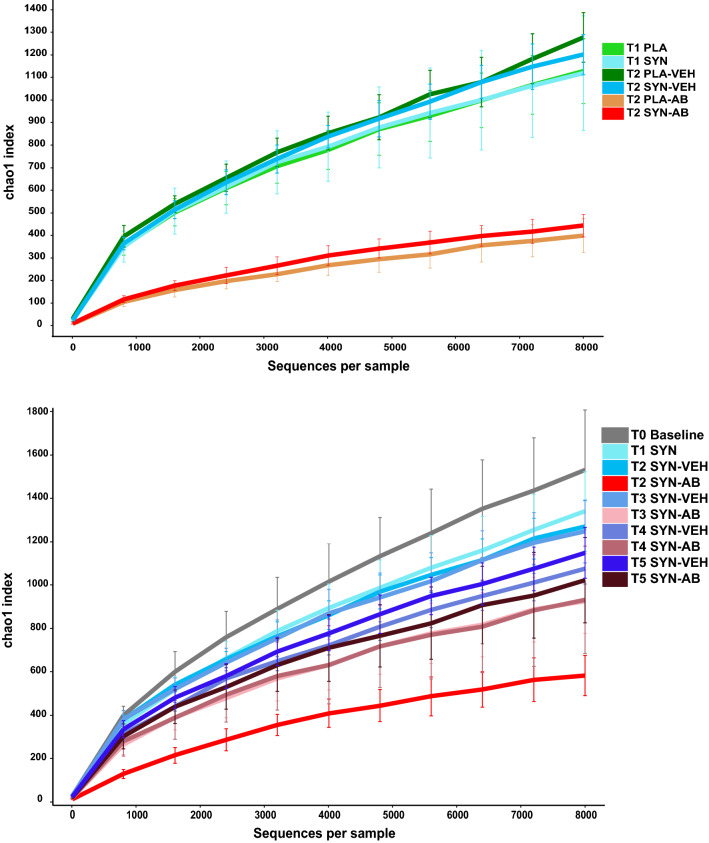
Alpha-rarefaction curves using the Chao1 index as a measure of bacterial richness in the samples obtained in protocol 1 (upper panel) and protocol 2 (lower panel). T0–T5 mark the time points when fecal pellets were harvested for microbiome analysis (see Figs. 1, 2). Treatments: PLA, placebo (for synbiotic); SYN, synbiotic; VEH, vehicle (for antibiotic treatment); AB, antibiotic combination

Protocol 1 was designed to examine microbiome changes after pretreatment with the synbiotic and subsequent antibiotic treatment (Fig. 1). Antibiotic treatment had a profound impact on the composition of the fecal microbiome as both bacterial diversity (beta diversity) (Fig. 3) and bacterial richness (alpha diversity) (Fig. 4, upper panel) were significantly reduced. Overall, the percentage of variance explained was rather high, amounting to 71.35% in the first component of principal coordinate analysis (PCoA) (Fig. 3), which attests to the efficacy of antibiotic treatment. The microbial communities in the feces of antibiotic-treated mice receiving placebo or synbiotic clustered rather close together and were clearly separated from those in vehicle-treated animals (*p* < 0.001 by Adonis test). In contrast, there was little separation between vehicle-treated mice receiving placebo or synbiotic (Fig. 3). Bacterial richness was decreased in both placebo- and synbiotic-treated mice which underwent antibiotic treatment (Fig. 4, upper panel).

### Effect of antibiotic treatment in the prolonged presence of the synbiotic in protocol 2

**Fig. 5:**
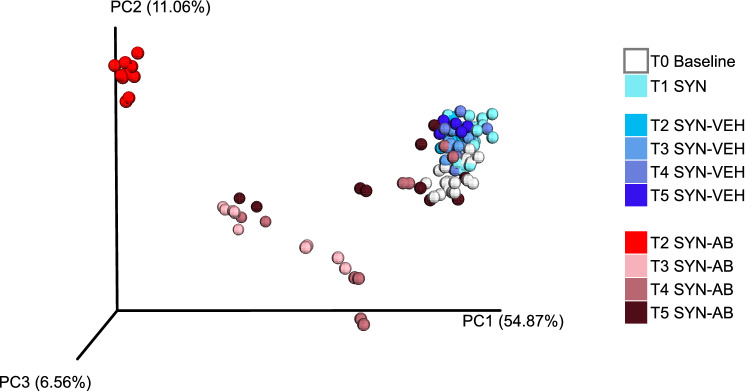
Principal coordinate analysis (PCoA) plot based on weighted UniFrac distance between samples obtained in protocol 2. T1–T5 mark the time points when fecal pellets were harvested for microbiome analysis (see Fig. 2). Treatments: SYN, synbiotic; VEH, vehicle (for antibiotic treatment); AB, antibiotic combination

Protocol 2 was designed to examine microbiome changes following antibiotic treatment in the prolonged presence of the synbiotic (Fig. 2). The pertinent data disclosed distinct time courses in the alterations of the fecal microbiome with regard to bacterial richness (alpha diversity) (Fig. 4, lower panel) and bacterial diversity (beta diversity) (Fig. 5). Relative to baseline (time point T0 before any treatment was begun), the bacterial community in synbiotic-treated animals receiving the vehicle did not change sufficiently during the time course of the experiment (> 16 weeks, time points T1–T5) to allow for a time-dependent separation in the PCoA plot (Fig. 5). In contrast, antibiotic treatment resulted in a clear clustering and separation of the microbiota profile at time point T2 (end of antibiotic treatment). Three weeks after the end of antibiotic treatment (T3), the microbial composition was appreciably shifted (light pink color) but had not yet returned to baseline levels (Fig. 5). An inconsistent further shift towards baseline levels was observed 9 weeks post-antibiotic treatment (T4, dark pink color). Twelve weeks post-antibiotic treatment (T5, dark red color), the microbial profile of the feces in two out of ten antibiotic-treated mice had returned to baseline values, whereas the microbial composition in the feces of the other eight mice remained in an intermediate cluster (Fig. 5).

### Effects of synbiotic and antibiotic treatment on distinct bacterial orders in protocol 1

**Fig. 6:**
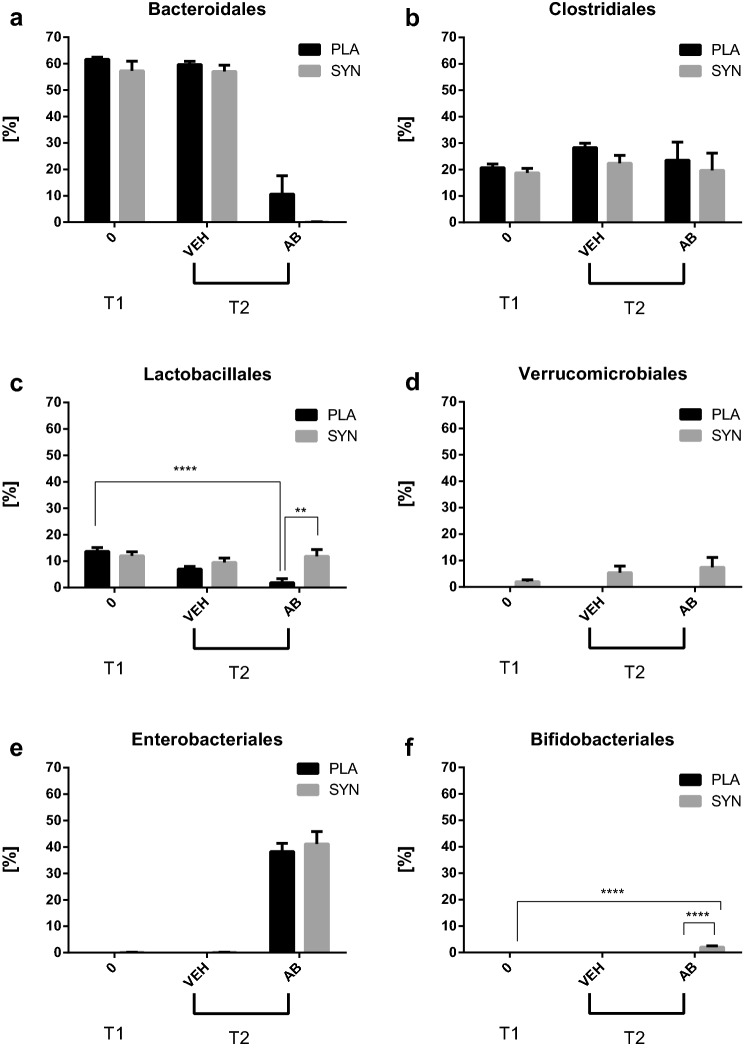
Relative abundances of bacterial orders in the fecal pellets collected at time points T1 and T2 of protocol 1 (see Fig. 1). The values represent means + SEM, *n* = 19 at T1, *n* = 9–10 at T2; **p < 0.01, ****p < 0.0001, comparisons as indicated (two-way ANOVA followed by Tukey’s HSD post hoc test). Treatments: PLA, placebo (for synbiotic); SYN, synbiotic; VEH, vehicle (for antibiotic treatment); AB, antibiotic combination

Although synbiotic treatment before and during antibiotic treatment had no gross effect on antibiotic-induced gut dysbiosis in protocol 1 (Figs. 3, 4), LEfSe analysis of bacterial orders present in the fecal pellets revealed distinct differences between placebo- and synbiotic-pretreated animals. Time- and treatment-related shifts in bacterial orders were statistically evaluated if the relative abundance of a bacterial order reached at least 2% in any of the samples. Lower taxonomic levels were not analyzed because not all OTU sequences could be classified at the family, genus and species levels. As shown in Fig. 6a, antibiotic treatment led to a nearly complete deletion of the order Bacteroidales in the placebo- and particularly in the synbiotic-pretreated mice. While the order of Clostridiales was virtually not modified by any of the treatments undertaken (Fig. 6b), the order of Lactobacillales was markedly reduced in the antibiotic-treated group receiving placebo, an effect that was prevented by the synbiotic (Fig. 6c).

The order of Verrucomicrobiales was detected to an appreciable extent only when the animals were pretreated with the synbiotic, independently of whether they were treated with vehicle or antibiotic (Fig. 6d). In contrast, the order of Enterobacteriales appeared prominently only in the mice undergoing antibiotic treatment, and this antibiotic effect remained unchanged by synbiotic pretreatment (Fig. 6e). The abundance of Rickettsiales and Streptophyta was likewise enlarged during antibiotic treatment in a synbiotic-independent manner (data not shown). In contrast, an appreciable number of Bifidobacteriales was observed only when antibiotic-treated animals had undergone synbiotic pretreatment (Fig. 6f).

### Effects of antibiotic treatment in the prolonged presence of the synbiotic on distinct bacterial orders in protocol 2

**Fig. 7:**
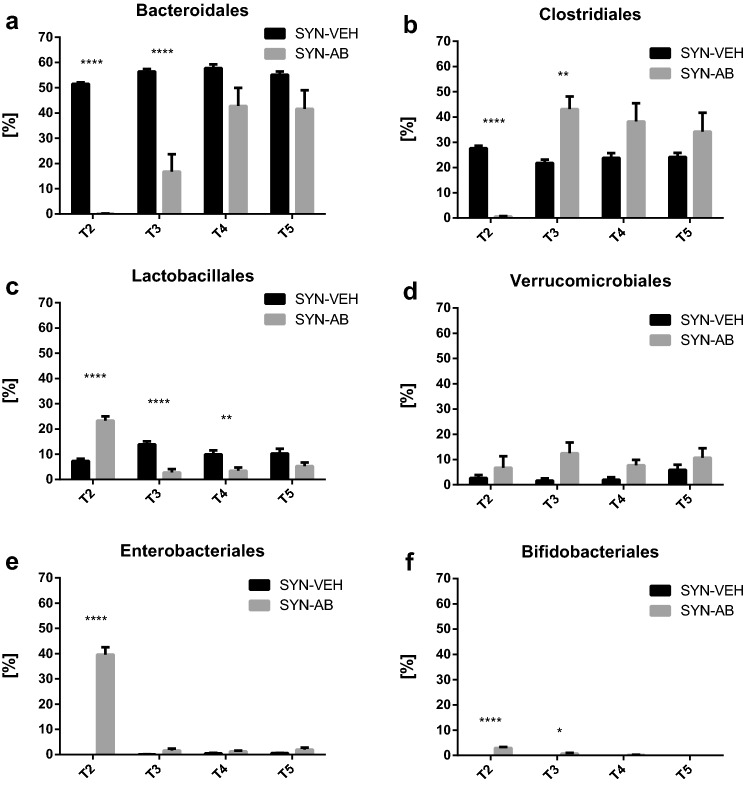
Relative abundances of bacterial orders in the fecal pellets collected at time points T2–T5 of protocol 2 (see Fig. 2). The values represent means + SEM, *n* = 10; *p < 0.05, **p < 0.01, ****p < 0.0001, SYN-AB vs. SYN-VEH at each time point (two-way repeated measures ANOVA followed by Sidak’s multiple comparison test). Treatments: SYN, synbiotic; VEH, vehicle (for antibiotic treatment); AB, antibiotic combination

Distinct alterations in the microbial profile were observed when the fecal pellets obtained in protocol 2 were analyzed at the level of bacterial orders. Whilst vehicle administration to synbiotic-treated animals did not modify the bacterial abundance to any gross extent during the course of the study (data not shown), antibiotic treatment followed by continued presence of the synbiotic altered the abundance of particular bacterial orders to a marked extent (Fig. 7). Thus, a pronounced depletion of Bacteroidales (Fig. 7a) and Clostridiales (Fig. 7b) was seen by the end of the antibiotic treatment period (T2). Three (T3), 9 (T4) and 12 weeks (T5) post-antibiotic treatment the order of Bacteroidales underwent a partial recovery in a time-dependent manner in the continued presence of the synbiotic (Fig. 7a).

In contrast, the recovery of Clostridiales was boosted in antibiotic/synbiotic-treated animals, as the abundance of Clostridiales at T3 already exceeded that seen in vehicle/synbiotic-treated mice (Fig. 7b). The relative abundances of Lactobacillales, Bifidobacteriales and Enterobacteriales and, to some extent, Verrucomicrobiales were also enhanced at completion of antibiotic treatment in the presence of the synbiotic (T2) (Fig. 7c–f). With the exception of Verrucomicrobiales, this change was not maintained during the following 12 weeks. While appreciable levels of Bifidobacteriales and Enterobacteriales were detected only at T2 in antibiotic-treated mice (Fig. 7e, f), the relative levels of Lactobacillales in antibiotic/synbiotic-treated mice at T3–T5 were in fact markedly lower than in vehicle/synbiotic-treated mice receiving the synbiotic and did not recover during the 12-week post-antibiotic period (Fig. 7c). In contrast, the relative abundance of Verrucomicrobiales in antibiotic/synbiotic-treated mice exceeded that in vehicle/synbiotic-treated animals during time points T2–T5 (Fig. 7d).

## Discussion

Analysis of the effects of antibiotic and synbiotic treatment on the murine fecal microbiome in two complementary experimental protocols revealed unexpected interactions between the two interventions. In particular, the impact of antibiotics on distinct bacterial orders is modified by concomitant synbiotic treatment in a time-dependent manner. Specifically, the effects seen during short-term exposure to antibiotics after synbiotic pretreatment are profoundly different from those seen following prolonged synbiotic administration in the post-antibiotic period. For instance, synbiotic pretreatment preserved the Lactobacillales population and boosted the relative abundance of Bifidobacteriales during antibiotic treatment. This effect was completely lost during continued synbiotic administration, despite the fact that both *Lactobacillus* and *Bifidobacterium* strains were components of the synbiotic administered. In fact, the relative abundance of Lactobacillales measured in antibiotic/synbiotic-treated animals fell below that seen in vehicle/synbiotic-treated mice during the post-antibiotic period. In contrast, the beneficial effect of synbiotic pretreatment on the Verrucomicrobiales during antibiotic treatment was maintained under prolonged synbiotic administration, while the relative abundance of Clostridiales was enhanced only with some delay in the post-antibiotic period under synbiotic treatment.

Diversity in its composition enables the gut microbiota to resist adverse changes in its environment (resistance) and to return to equilibrium following perturbation (resilience) [4]. In the present study, we specifically challenged the gut microbiota by oral administration of a combination of non-absorbable antibiotics consisting of four compounds with a different mechanism and spectrum of antibacterial action. The ability of this antibiotic combination plus ampicillin to deplete the gut microbiota has previously been demonstrated [40]. Ampicillin was excluded from the combination used here, because it is absorbed from the gut [40] and could have confounding systemic effects. By targeting a broad spectrum of the bacterial components of the gut microbiota, we wanted to create a niche in which the synbiotic strains could proliferate and interact with the gut microbiota more profoundly than under physiologic conditions. Several studies have shown that, under physiologic or IBS-D conditions, pro- and synbiotics can colonize the human large intestine only to a very limited extent [7, 33, 34, 46].

In experimental protocol 1, we examined how the microbial community is affected by gut-directed antibiotic treatment and analyzed the resistance of the microbial community in response to antibiotic and synbiotic challenge. Although pro- and synbiotics are in general thought to ameliorate the adverse effect of antibiotics on the gut microbiota [26, 29–32], this view has been questioned by a report that probiotics colonizing the gut may rather impair than aid the recovery of the gut microbiota after antibiotic treatment [7]. For this reason, we assessed the effect of a gut-directed antibiotic treatment under prolonged synbiotic treatment in experimental protocol 2. With these two approaches, we aimed at obtaining a deeper insight into the mutual interaction between antibiotics and synbiotics in shaping the architecture of the gut microbiota.

Placebo- and synbiotic-treated animals did not appreciably differ in bacterial richness and diversity from each other, which is in keeping with the reported resistance of the murine intestinal microbiota to probiotic colonization [46]. In contrast, antibiotic treatment caused microbial depletion as deduced from a marked decrease in bacterial richness and diversity, confirming a previous report [40]. However, analysis of the fecal microbiome at the order level, at which we were able to assign all sequences measured, revealed that some distinct taxa were increased in relative abundance following exposure to the antibiotics. Thus, the relative abundance of Enterobacteriales reached a maximum at the end of the antibiotic treatment period in both protocol 1 and 2 and dwindled quickly thereafter, which is in keeping with the outcome of other experimental studies [47–50]. A similar enlargement of the Enterobacteriales population has been observed 7 days after infection of mice with *Citrobacter rodentium* when gastrointestinal inflammation has become manifest [51].

Antibiotic treatment likewise enhanced the relative abundance of Lactobacillales and Bifidobacteriales as noted at the end of the antibiotic treatment period in protocols 1 and 2. Since this antibiotic effect was observed only following synbiotic pretreatment, it is conceivable that it reflects intestinal colonization of Lactobacillales and Bifidobacteriales strains present in the synbiotic. However, this argument is questioned by the observation that the increase in relative abundance of Lactobacillales and Bifidobacteriales was not maintained during the post-antibiotic period despite the continued administration of the synbiotic. We hypothesize that changes in the microbiome architecture during the post-antibiotic period occluded the ecologic niche in which the synbiotic-derived Lactobacillales and Bifidobacteriales found a favorable habitat to colonize during antibiotic treatment. This contention is supported by similar findings reported by Suez et al. [7] during treatment of mice with a combination of ciprofloxacin and metronidazole concomitantly with an 11-strain probiotic including *Lactobacillus* and *Bifidobacterium* species. The dynamic alterations in Lactobacillales and Bifidobacteriales abundance during and after antibiotic treatment in the presence of a multi-strain synbiotic as found here is likely to have a bearing on other components of the microbiota, since members of the Lactobacillales and Bifidobacteriales order are known to modulate the activity and vitality of other gut microbes [52–56]. Specifically, soluble factors secreted from *Lactobacillus* are able to inhibit the growth of the human fecal microbiota in vitro by reducing the number of observed species and modulating the community structure [7].

Another bacterial taxon that specifically increased in relative abundance following combined synbiotic and antibiotic treatment is the order of Verrucomicrobiales, specifically *Akkermansia muciniphila*, the sole identified member of this order [57, 58]. The marked rise of *Akkermansia* abundance seen at the end of the antibiotic treatment period was maintained when synbiotic administration was continued during the post-antibiotic period. As in the case of Lactobacillales and Bifidobacteriales, antibiotic treatment appeared to clear a niche in which the synbiotic could induce *Akkermansia* to proliferate for a prolonged period of time. This finding may be of physiological significance, given that *Akkermansia* is a mucin-degrading bacterium that resides in the mucus layer of the gut [57], strengthens intestinal barrier integrity and regulates gut metabolism [23, 58]. In view of this biological profile, *Akkermansia muciniphila* is thought to be a candidate probiotic with a potential benefit in pathologies such as obesity, diabetes, liver injury and cardiovascular disorders [23, 58, 59]. The abundance of *Akkermansia muciniphila* can also be increased by prebiotic supplementation with oligofructose although, in vitro, *Akkermansia muciniphila* does not grow on oligofructose-enriched media [57]. The synbiotic used here contains prebiotically active compounds such as maltodextrin, inulin and fructooligosaccharides, maltodextrin being also present in the placebo. It should therefore not go unnoticed that these compounds may contribute to the effects of the synbiotic on the intestinal microbiota.

Analysis of the post-antibiotic period revealed that the fecal microbiome did not fully recover in the continued presence of the synbiotic. This outcome needs to be seen in conjunction, on the one hand, with the reported resistance of the murine intestinal microbiota to probiotic colonization [46] and, on the other hand, with the reported ability of probiotics to delay post-antibiotic gut microbiota recovery [7]. Nevertheless, cessation of antibiotic treatment resulted in an increase in bacterial diversity first analyzed here 3 weeks post-antibiotic treatment, which is in overall agreement with a partial restoration of gut microbiota composition already 1 week after the end of ampicillin, streptomycin and clindamycin administration [60]. However, during the period between 3 and 9 weeks post-antibiotic treatment, the recuperation of bacterial richness and diversity proceeded at a low speed. At the taxonomic level, the microbial community stayed deficient in the order of Lactobacillales while the order of Bacteroidales underwent a delayed but incomplete recovery. In contrast, we saw a maintained enlargement of the Clostridiales order in the post-antibiotic period under synbiotic administration. Analogous results have been reported following treatment of mice with a combination of ampicillin, vancomycin, metronidazole and neomycin [47].

These findings illustrate the dynamics of the intestinal microbial ecosystem that comes to light when its integrity is disturbed by antibiotic treatment. In analyzing the complex interactions that we observed between the synbiotic and antibiotic treatment, a number of limitations need be considered. These interactions will depend on the antibiotic sensitivity of particular bacterial taxa in the native microbiota and in the synbiotic as well as on a multitude of positive and negative interactions between distinct taxa of the gut microbiota and the synbiotic. The interactions may also be influenced by factors of the host organism, which we wanted to avoid to a certain extent by using only non-absorbable antibiotics. In interpreting the observed changes in bacterial taxa, it needs to be taken into account that alterations of the relative abundance of a given taxon will increase or decrease if the relative abundance of other taxa decreases or increases, respectively. Finally, it needs to be borne in mind that the effects of the synbiotic under study may be the net effect of its pre- and probiotic ingredients, whose contributions remain to be analyzed.

In conclusion, our data show that the synbiotic under study per se had an insignificant influence on the microbial community but was able to modulate the impact of antibiotic treatment on the fecal microbiota in a time-dependent fashion because the antibiotic-induced microbial perturbations underwent distinct changes when the administration of the synbiotic was continued during the post-antibiotic period. The pertinent observations disclose dynamic interactions between synbiotic and antibiotic interventions in shaping the intestinal microbiota. These interactions need to be construed in light of the resistance and resilience of the microbial community to challenges of its integrity. In addition, it is important to bear in mind that the baseline composition of the intestinal microbiota is a factor that determines colonization of the gut by bacterial components [46] of the synbiotic. Apart from their beneficial effects on host physiology, certain components of the synbiotic such as *Lactobacillus* can also secrete molecules that inhibit microbial proliferation [7]. These considerations prompt the conclusion that potential benefits of synbiotics per se and under antibiotic treatment cannot be predicted without knowledge of the individual conditions determining microbiota, synbiotic and antibiotic interactions. It is very likely that a lack of knowledge of these conditions is one of the major reasons why the outcomes of clinical synbiotic trials are frequently inconsistent and inconclusive [20, 61, 62].
